# Endophytic fungus *Serendipita indica* accelerates ascorbate-glutathione cycle of white clover in response to water stress

**DOI:** 10.3389/fmicb.2022.967851

**Published:** 2022-08-01

**Authors:** Zi-Yi Rong, Dao-Ju Jiang, Jin-Li Cao, Abeer Hashem, Elsayed Fathi Abd_Allah, Mashail Fahad Alsayed, Wiwiek Harsonowati, Qiang-Sheng Wu

**Affiliations:** ^1^College of Horticulture and Gardening, Yangtze University, Jingzhou, China; ^2^Shashi Substation, Jingzhou Municipal Bureau of Natural Resources and Planning, Jingzhou, China; ^3^Department of Botany and Microbiology, College of Science, King Saud University, Riyadh, Saudi Arabia; ^4^Plant Production Department, College of Food and Agricultural Sciences, King Saud University, Riyadh, Saudi Arabia; ^5^Department of Agrobiology and Bioresources, School of Agriculture, Utsunomiya University, Utsunomiya, Japan

**Keywords:** ascorbic acid, fungi, glutathione, reactive oxygen species, water stress

## Abstract

Ascorbate-glutathione cycle is an important pathway for plants to scavenge reactive oxygen species (ROS) under environmental stress conditions. The objective of this study was to investigate the effects of the endophytic fungus *Serendipita indica* on biomass, chlorophyll concent, ROS levels, antioxidant enzyme activities, and ascorbate-glutathione cycle in white clover under ample water and water stress conditions. The results showed that 46 days of soil water stress distinctly promoted root colonization by *S. indica*. Under water stress, *S. indica* inoculation significantly promoted shoot and root biomass, total chlorophyll content, and activities of superoxide dismutases (SOD; e.g., Fe-SOD and Cu/Zn-SOD) and peroxidase in roots, coupled with a decrease in malondialdehyde content in roots. In the ascorbate-glutathione cycle of roots, *S. indica* also significantly increased the activity of ascorbate peroxidase and glutathione reductase activities in water-stressed white clover, along with the increase in reduced ascorbic acid and reduced/oxidized glutathione contents, thus accelerating the ascorbate-glutathione cycle in inoculated plants to scavenge more ROS (e.g., hydrogen peroxide). As a result, *S. indica* enhanced the tolerance of white clover in response to water stress by enhancing antioxidant enzyme activities and accelerating the ascorbate-glutathione cycle.

## Introduction

White clover (*Trifolium repens* L.) is a common perennial legume plant, which is often used in temperate regions for greening and soil conservation because of its strong expansion ability and fast regeneration rate. However, white clover favors wet and cold environments, and deficiency of water seriously affects its growth and functions (Liang et al., 2021).

Water stress (WS) is one of environmental stresses limiting plant growth and has a negatively impact on physiological activities of plants, especially on the production of reactive oxygen species (ROS) in cells, which can cause serious damage to plant tissues (Cheng et al., 2021; Zou et al., 2021a). Plants also improve their adaptation to drought by strengthening their antioxidant defense system including ascorbate-glutathione cycle (Cao et al., 2022). Ascorbate peroxidase (APX), ascorbic acid (ASC), glutathione reductase (GR), and glutathione (GSH) belong to ascorbate-glutathione cycle, which is an essential pathway for plants to avoid oxidative burst (Cengiz et al., 2020). The cycle is a scavenging pathway of H_2_O_2_ by modulating glutathione/glutathione disulfide (GSH/GSSG) and ascorbate/dehydroascorbate (ASC/DHA) interconversion, which primarily operates in chloroplast but also found in other parts of cytosol such as in mitochondria and peroxisomes (Li et al., 2022a). ASC and GSH are used primarily to remove hydrogen peroxide (H_2_O_2_) in ROS. ASC can directly quench H_2_O_2_ or act as the substrate of APX to catalyze the reduction of H_2_O_2_. GSH, as the electron donor of monodehydroascorbate reductase/dehydroascorbate reductase, regenerates ASA by reducing the product monodehydroascorbic acid/dehydroascorbic acid catalyzed by APX. GR is responsible for the recovery of GSH (Cao et al., 2021). Hence, ASC and GSH are present in plant cells, mostly in their reduced form, and they are able to detoxify oxidative damage under stress conditions (Iwona et al., 2021).

Previous studies have found that a beneficial root endophytic fungus, *Serendipita indica*, enhanced the activity of antioxidant enzymes and thus improved the resistance of plants exposed to WS (Aslam et al., 2019). *Serendipita indica* has functions similar to those of arbuscular mycorrhizal fungi. Unlike arbuscular mycorrhizal fungi, *S. indica* can be proliferated *in vitro* through the artificial medium without plant roots. *Serendipita indica* exists as pear-shaped chlamydospores within and between the cortical cells of roots (Khalid et al., 2022). This fungus improves plant growth, promotes nutrient absorption, and enhances stress resistance of plants (Aslam et al., 2019). It has been shown that inoculation of *S. indica* on *Solanum melongena* plants significantly increased peroxidase (POD) and catalase (CAT) activities in roots under WS (Swetha and Padmavathi, 2019). *Serendipita indica* also increased CAT and GR activities but reduced GSH content of rice under WS, along with no change in superoxide dismutase (SOD) and APX activities and reduced ASC content (Tsai et al., 2020). In a study performed by Li et al. (2022b), inoculation of *S. indica* in the field did not induce expression levels of *CsFe-SOD* and *CsMn*-*SOD* in leaves of Lane Late navel orange, but stimulated *CsPOD* and *CsCAT1* expression levels, along with an inhibited expression of *CsCu/Zn-SOD*. In rice, APX and ASC were not induced by *S. indica* under WS (Tsai et al., 2020), suggesting that the mechanism underlying the effect of *S. indica* on the host ascorbate-glutathione cycle is still complex.

However, does *S. indica* enhance the tolerance of white clover under WS? Is this increased tolerance associated with an enhanced ascorbate-glutathione cycle? To answer the two questions, the aim of this study was to analyze the effects of *S. indica* inoculation on the growth, ROS levels, antioxidant enzyme activities, and ascorbate-glutathione cycle of white clover under normal water (NW) and WS.

## Materials and methods

### Fungal inocula

*Serendipita indica* was provided by Prof. Zhi-Hong Tian, Yangtze University. *Serendipita indica* pieces were inoculated into solid potato glucose medium at 23°C for 3 weeks. After the mycelium was full of the medium, a small amount of sterilized distilled water was added. Sterile glass rod was used to scrape the fungus from the medium surface and put it in a triangular flask with a constant volume of 500 ml ddH_2_O to obtain the spore suspension of *S. indica*. In addition, *S. indica* pieces were also inoculated in liquid potato glucose medium in the dark culture for 7 days at 30°C and 150 × *g*/min. The mycelia were washed with ddH_2_O for three times and filtered. The 1 g of fresh mycelia was mixed with 100 ml ddH_2_O to obtain mycelia liquid of *S. indica*.

### Plant culture

Fifteen seeds of white clover from Hubei Seed Station were planted in a plastic pot (16 cm × 11 cm × 13 cm), where 1,200 g of growth substrate has been supplied. The potted substrate was the mixture with soils and river sand (3:1, v/v) and autoclaved. *S. indica* inoculation was carried out at the time of sowing, in which each pot of inoculation treatment received 14.5 ml mycelium liquid and 12.5 ml spore suspension. Non-inoculation with *S. indica* received equal amounts of autoclaved mycelium liquid and spore suspension. Subsequently, soil moisture of these plants was controlled under NW conditions. Plants were placed in a controlled environment described by Zou et al. (2021b).

After 2 weeks, the seedlings were thinned to 12 plants per pot. Four weeks later, WS was started. One half of the inoculated and uninoculated plants were randomly selected, and the soil water regime was adjusted to WS, while the other half remained at NW. The soil moisture content was determined by weighing at 18:00 every day, and the water lost was replenished daily in time to maintain the soil moisture content of the earlier design for each pot. No any fertilizer was added during the experiment.

### Experimental design

This experiment consisted of four treatments: (1) white clover was inoculated with *S. indica* under NW (75% of the maximum field water capacity; NW + Si); (2) white clover was inoculated without *S. indica* under NW (NW-Si); (3) white clover was inoculated with *S. indica* under WS (50% of the maximum field water capacity; WS + Si); and (4) white clover was inoculated without *S. indica* under WS (WS-Si). Each treatment replicated five times, and a total of 20 pots were randomly arranged.

### Measurements of plant growth, chlorophyll concentrations, and root fungal colonization rate

After 46 days of WS treatment, the experiment has ended, and the biomass of shoots and roots was weighed. Chlorophyll concentrations were assayed using the method of Ren et al. (2014) with the extraction of 80% of acetone solutions. Root fungi were stained as per the procedure of Phillips and Hayman (1970). The 1-cm-long root segments were incubated in 10% KOH at 90°C for 50 min, bleached by 10% H_2_O_2_ for 15 min, acidified by 0.2 mol/L HCl for 10 min, and stained by 0.05% trypan blue in lactophenol for 30 s. Fungal colonization was observed microscopically in the roots, and root fungal colonization rate was calculated by the formula of Yang et al. (2021).

### Measurements of root ROS and MDA concentrations

The content of hydrogen peroxide (H_2_O_2_) in roots was determined by the method described by Velikova et al. (2000), in which 2 ml of the reaction contained 0.5 ml supernatant, 0.5 ml of 10 mmol/L potassium phosphate buffer (pH 7.0), and 1 ml of 1 mol/L KI solution. The content of superoxide anion radical (O_2_^•−^) in roots was determined according to the method described by Li et al. (2022b). The concentration of malondialdehyde (MDA) was determined by Sudhakar et al. (2001).

### Measurements of antioxidant enzyme activities in roots

The 0.3 g of roots was ground with 7 ml of pre-cooled 0.1 mol/L phosphate buffer (pH 7.8) and centrifuged at 4,000 × *g* for 15 min. The supernatant was used for the analysis of CAT and POD activities in roots. Among them, CAT activity was determined by potassium permanganate titration (Wu, 2018). POD activity was determined by guaiacol method (Wu, 2018), in which 5 ml of the reaction consisted of 2.9 ml of 0.05 mol/L phosphate buffer, 1 ml of 2% H_2_O_2_, 1 ml of 0.05 mol/L guaiacol, and 0.1 ml supernatant. Activity of APX in roots was assayed according to the procedure outlined by Li et al. (2022b), where a reaction solution with a total volume of 2.1 ml consisted of 0.1 ml 0.06% H_2_O_2_, 1.7 ml of 50 mmol/L potassium phosphate buffer containing 0.1 mmol/L EDTA-Na_2_, 0.1 ml of 6 mmol/L ASC, and 0.1 ml supernatant. Activity of GR in roots was determined as per the method of Fatemeh et al. (2020), in which 1.0 ml of the reaction mixture contained 50 mmol/L potassium phosphate buffer (pH 7.5), supernatant, 1 mmol/L GSSG, 0.75 mmol/L [5,5′-dithiobis-(2-nitrobenzoic acid)], and 0.1 mmol/L NADPH. The activities of Fe-SOD, Mn-SOD, and Cu/Zn-SOD in roots were determined by Enzyme-Linked Immunosorbent Assay, and the kits were purchased from Shanghai Enzyme-linked Biotechnology Co., Ltd.

### Measurements of antioxidant concentrations in roots

The 0.8 g root samples were ground with 5 ml 5% sulfosalicylic acid and centrifuged at 12,000 × *g* at 4°C for 25 min. ASC and DHA contents in the supernatant were determined according to the method described by Gao (2006). GSH and GSSG contents in the supernatant were determined according to the procedure described by Wu et al. (2006). The assay of GSH contained the supernatant, 100 mmol/L phosphate buffer, and dithiocarbenzoic acid (DTNB) at 30°C for 5 min, and the absorbance at 412 nm was recorded. In addition, total GSH mixture contained the supernatant, 100 mM phosphate buffer, one unit of GR, 0.15 mM NADPH, and DTNB at 30°C for 5 min, and the absorbance of the color solution at 412 nm. GSSG = total GSH—GSH.

### Statistical analysis

The SAS software was used for the ANOVA for the data. Duncan’s multiple range tests compared significant differences between treatments at the 0.05 level.

## Results

### Root colonization of *Serendipita indica*

**Figure 1:**
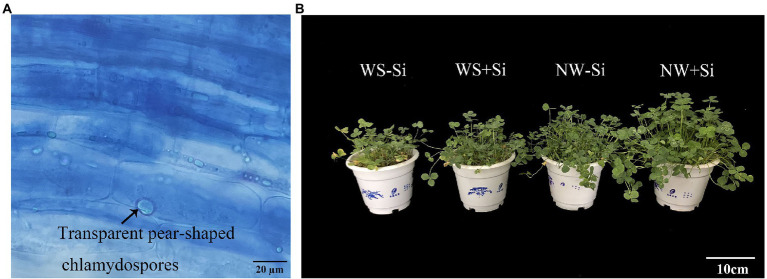
Root colonization of *Serendipita indica* (Si) **(A)** and plant growth responses **(B)** of white clover inoculated with Si under normal water (NW) and water stress (WS).

There was no evidence of fungal colonization in the roots of white clover without *S. indica* inoculation, while the root fungal colonization rate of *S. indica*-inoculated plants was 77.30% under NW to 92.56% under WS (Table 1). Soil WS treatment increased root colonization rate of *S. indica* by 19.74% (Figure 1A).

**Table 1:** Changes in root colonization rate, biomass, and total chlorophyll of white clover inoculated with *Serendipita indica* (Si) under normal water (NW) and water stress (WS).

Treatments	Root fungal colonization rate (%)	Shoot biomass (g/plant)	Root biomass (g/plant)	Total chlorophyll (mg/g)
NW-Si	0 ± 0c	30.74 ± 1.28b	8.83 ± 0.62b	1.38 ± 0.30c
NW + Si	77.30 ± 4.76b	35.13 ± 2.27a	9.97 ± 0.66a	2.25 ± 0.23a
WS-Si	0 ± 0c	14.09 ± 1.17d	5.21 ± 0.26d	1.42 ± 0.15c
WS + Si	92.56 ± 3.42a	16.98 ± 1.56c	6.11 ± 0.44c	1.87 ± 0.12b

Data (means ± SD, *n* = 5) followed by different letters in the column indicate significant differences (*p* < 0.05).

### Biomass production

Water stress treatment significantly suppressed the shoot and root biomass of inoculated and non-inoculated white clover, while *S. indica* significantly increased the shoot and root biomass by 14.28 and 12.91% under NW conditions, and by 20.51 and 17.27% under WS conditions, respectively (Figure 1B). In addition, the benefits of *S. indica* in promoting the shoot and root biomass were significantly higher under WS than under NW (Table 1).

### Total chlorophyll levels

The WS treatment significantly suppressed the total chlorophyll level of inoculated plants, but had no significantly impact on the total chlorophyll level of uninoculated plants (Table 1). *Serendipita indica* inoculation also significantly increased the total chlorophyll level under NW and WS conditions by 63.04 and 31.69%, respectively.

### ROS levels

**Figure 2:**
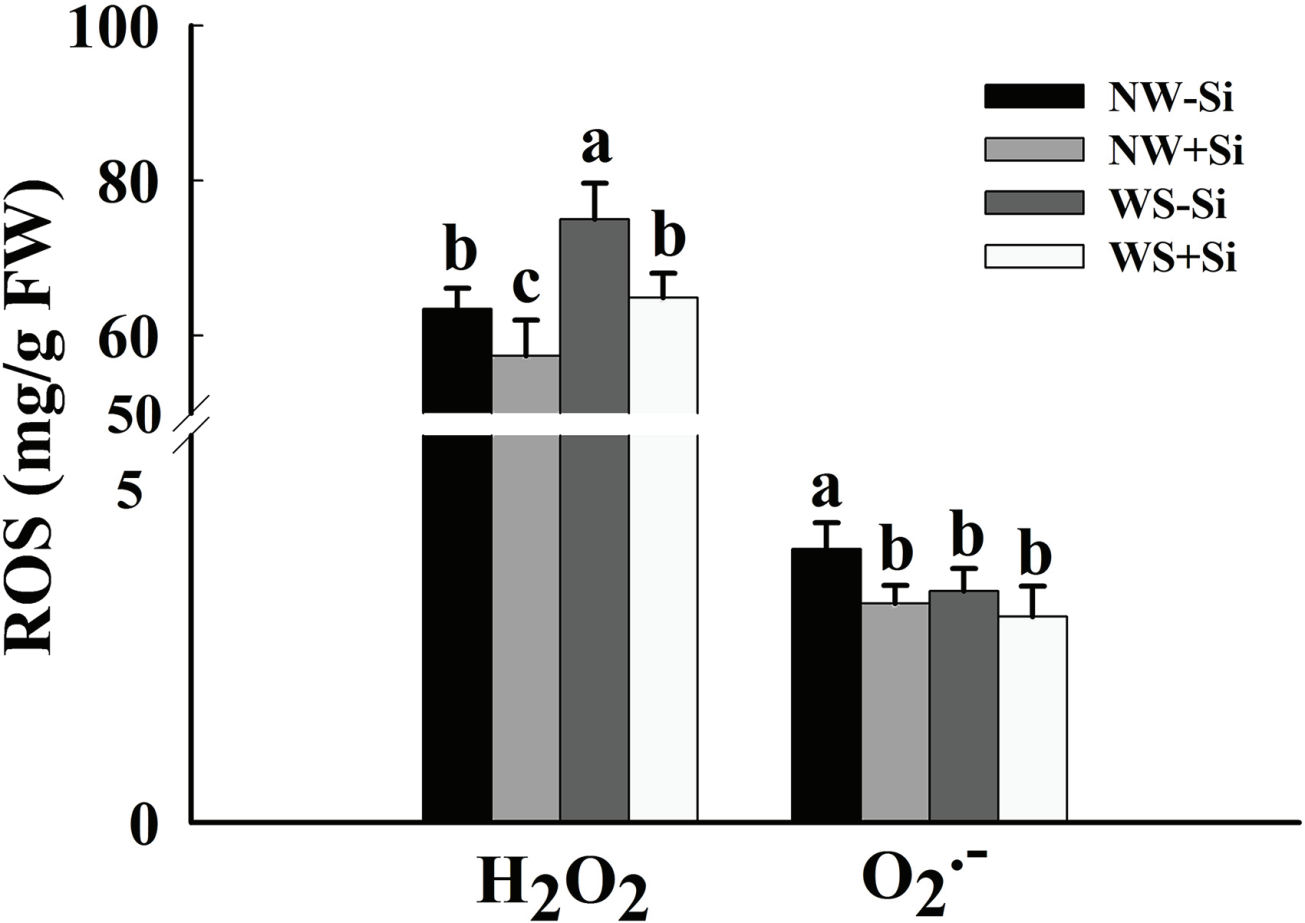
Changes in root H_2_O_2_ and O_2_^•−^ concentrations of white clover inoculated with *Serendipita indica* (Si) under normal water (NW) and water stress (WS). Data (means ± SD, *n* = 5) are significantly (*p* < 0.05) different if followed by different letters above the bars.

Water stress treatment significantly triggered an increase in H_2_O_2_ levels in roots of both inoculated and uninoculated plants, while suppressed O_2_^•−^ levels in roots of both inoculated and uninoculated plants (Figure 2). Inoculation with *S. indica* significantly reduced the root H_2_O_2_ levels by 9.46% under NW and by 13.54% under WS. Inoculation with *S. indica* only significantly reduced the root O_2_^•−^ levels by 19.83% under NW, coupled with no significant effect on root O_2_^•−^ levels under WS.

### MDA levels

**Figure 3:**
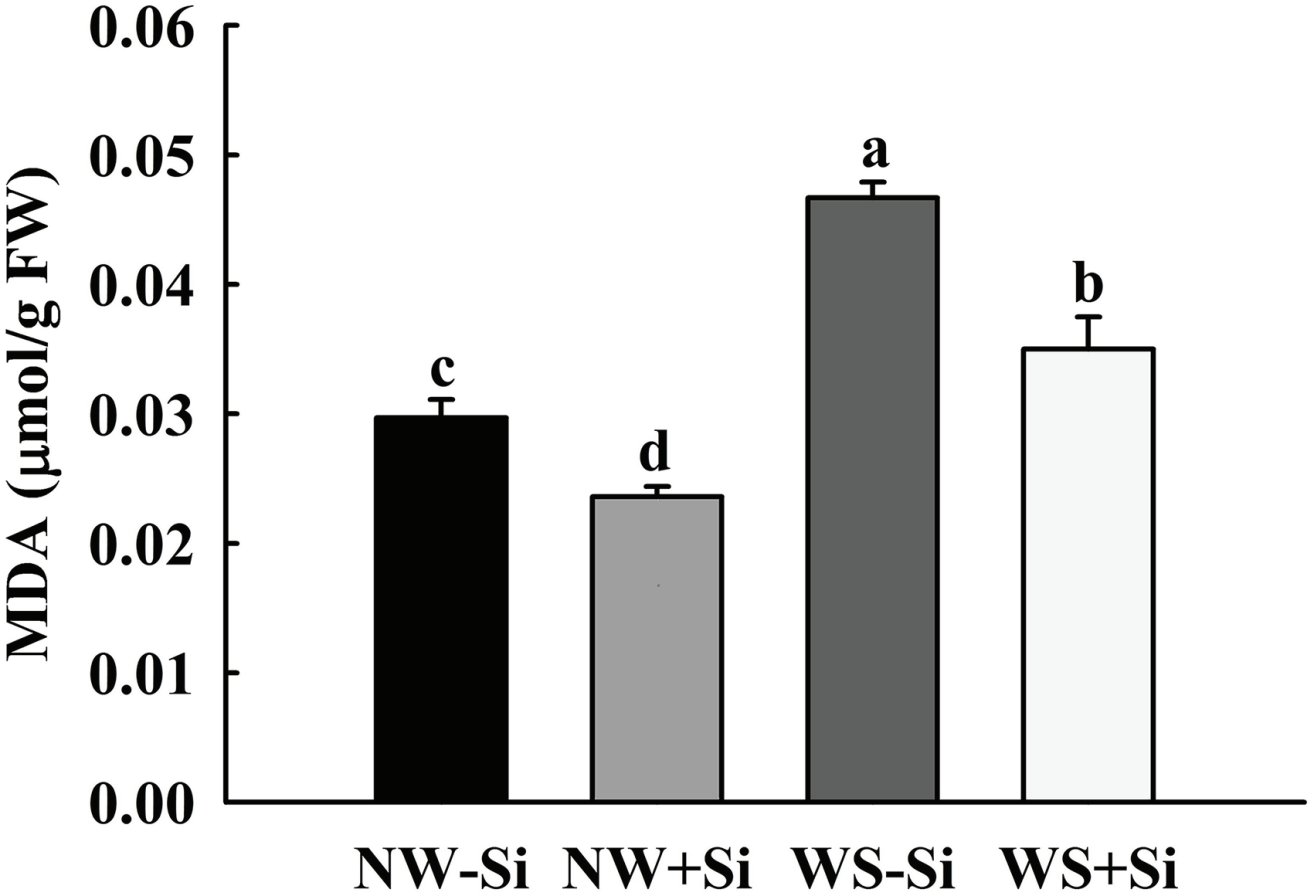
Changes in root malondialdehyde (MDA) concentrations of white clover inoculated with *Serendipita indica* (Si) under normal water (NW) and water stress (WS). Data (means ± SD, *n* = 5) are significantly (*p* < 0.05) different if followed by different letters above the bars.

The WS treatment induced an increase in the root MDA level by 36.40 and 32.57% in inoculated and uninoculated plants, respectively (Figure 3). In addition, inoculation of *S. indica* significantly reduced the root MDA level by 20.54 and 25.05% under NW and WS, respectively.

### Antioxidant enzyme activities

**Figure 4:**
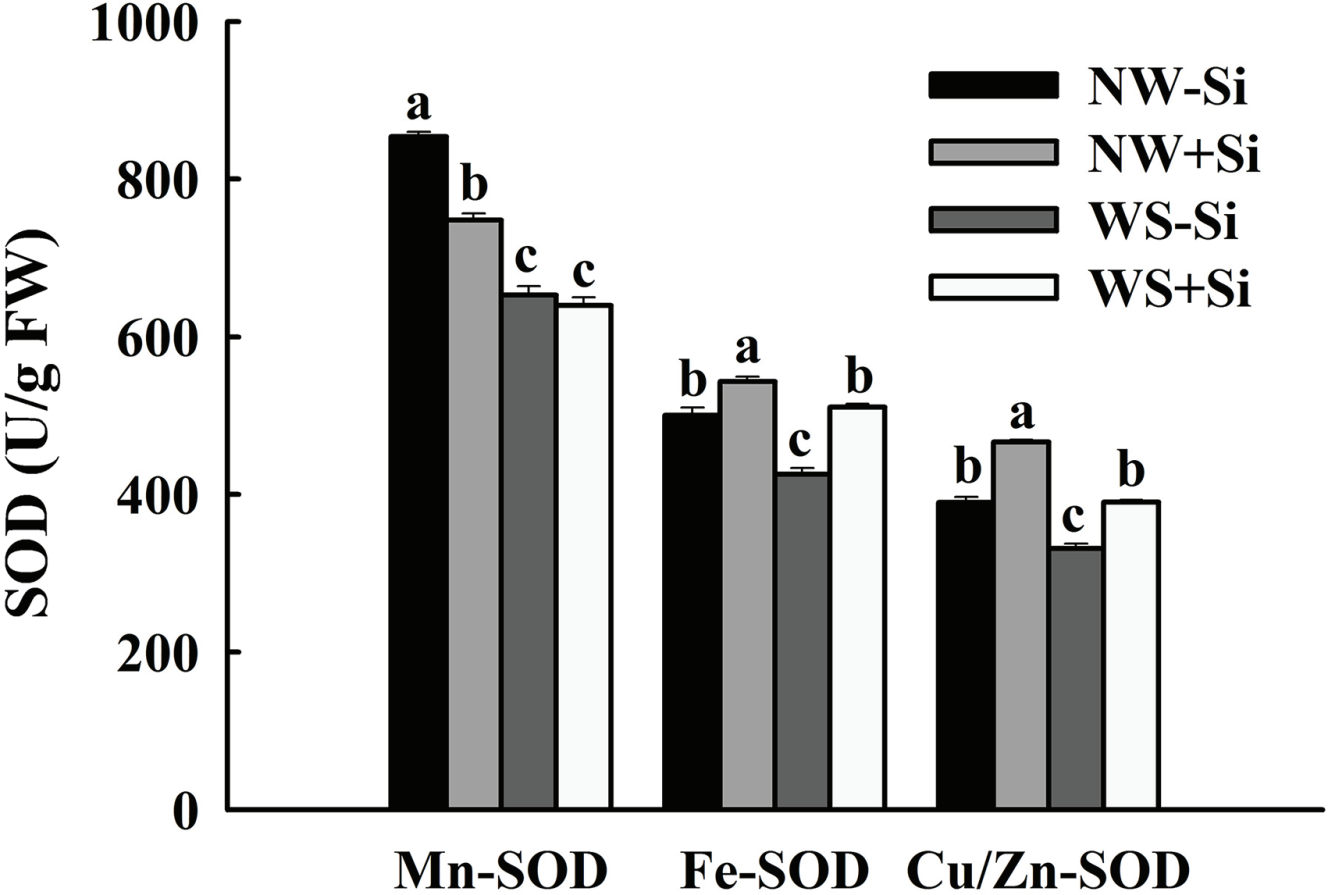
Changes in three superoxide dismutases (SODs) activities in roots of white clover inoculated with *Serendipita indica* (Si) under normal water (NW) and water stress (WS). Data (means ± SD, *n* = 5) are significantly (*p* < 0.05) different if followed by different letters above the bars.

Soil WS treatment significantly decreased the activities of Mn-SOD, Fe-SOD, and Cu/Zn-SOD in the roots of inoculated and non-inoculated plants (Figure 4). In addition, after inoculation under NW conditions, Fe-SOD and Cu/Zn-SOD activities increased significantly by 7.86 and 16.46%, respectively, while Mn-SOD activity decreased by 12.39%. Under the condition of WS, inoculation with *S. indica* could increase Fe-SOD and Cu/Zn-SOD activities by 16.71 and 15.07%, respectively, along with no significant effect on Mn-SOD activity.

**Figure 5:**
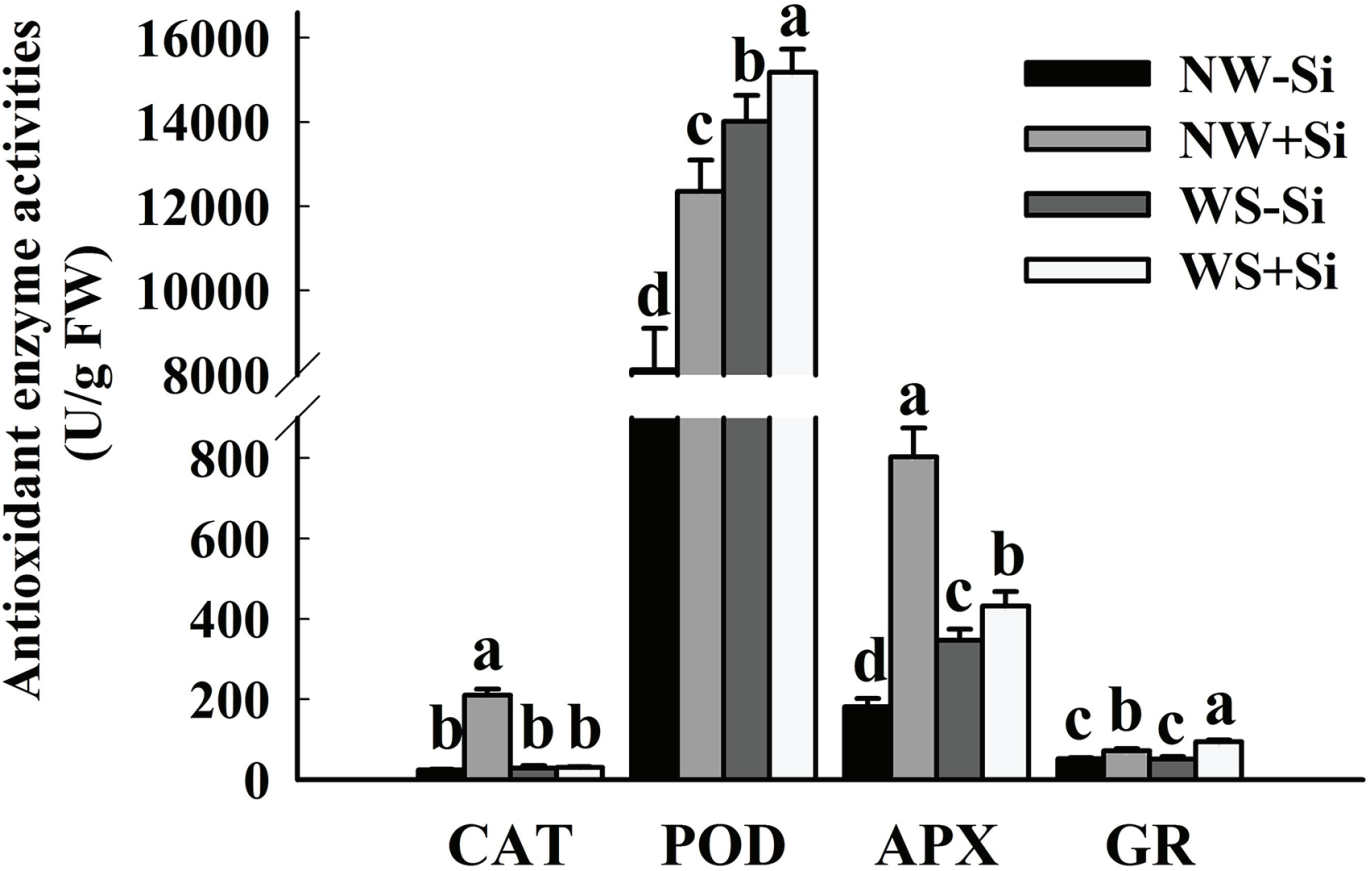
Changes in catalase (CAT), peroxidase (POD), ascorbate peroxidase (APX), and glutathione reductase (GR) activities in roots of white clover inoculated with *Serendipita indica* (Si) under normal water (NW) and water stress (WS). Data (means ± SD, *n* = 5) are significantly (*p* < 0.05) different if followed by different letters above the bars.

Soil WS treatment significantly reduced root CAT and APX activities in inoculated plants, while it increased root POD activity in inoculated and uninoculated plants, APX activity in uninoculated plants, and GR activity in inoculated plants (Figure 5). In addition, CAT, POD, APX, and GR activities were significantly increased by 88.39, 34.29, 77.34, and 28.07%, respectively, after inoculated with *S. indica* under AW. Under the condition of WS, inoculation with *S. indica* increased POD, APX, and GR activities by 7.66, 19.85, and 46.07%, respectively, with no significant effect on CAT activity.

### Antioxidant concentrations

**Figure 6:**
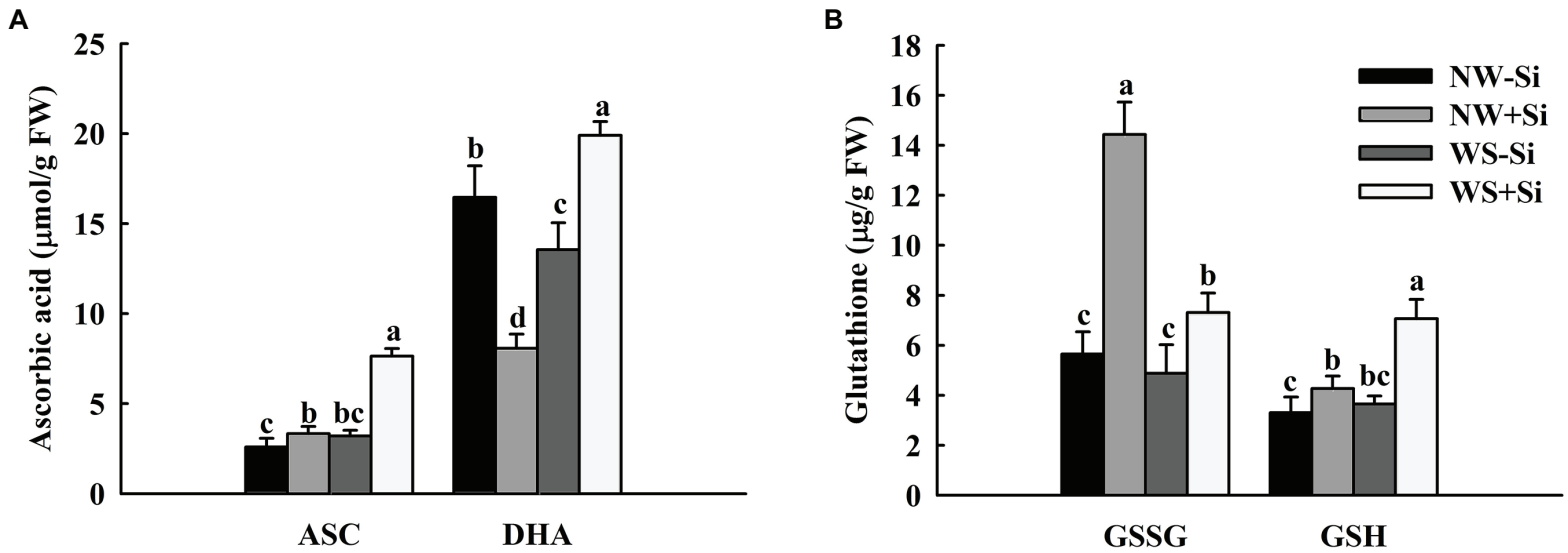
Changes in ascorbic acid **(A)** and glutathione **(B)** concentrations in roots of white clover inoculated with *Serendipita indica* (Si) under normal water (NW) and water stress (WS). Data (means ± SD, *n* = 5) are significantly (*p* < 0.05) different if followed by different letters above the bars.

The WS treatment induced a decrease of root DHA concentrations, with no significant effect on root ASC, GSH, and GSSG concentrations of uninoculated plants (Figures 6A,B). The root ASC, GSH, and DHA concentrations of inoculated plants increased significantly under WS, with a decrease in the root GSSG concentrations. In addition, the root ASC, GSH, and GSSG concentrations were significantly increased by 22.22, 22.72, and 60.85% under NW and by 58.06, 48.3, and 33.24% under WS after *S. indica* inoculation, respectively. Inoculation with *S. indica* significantly increased the root DHA concentrations by 31.86% under WS, but decreased it by 50.97% under NW.

## Discussion

The results of this study showed that WS had a positive effect on *S. indica* colonization of white clover roots. WS might trigger senescence of root cells, while mycelium of *S. indica* is found mainly in dead root epidermal and cortical cells (Deshmukh et al., 2006). Therefore, proper soil water deficit favors the colonization of roots by *S. indica*. The results of this study also showed that WS treatment significantly inhibited shoot and root biomass production in white clover, but *S. indica* colonization significantly enhanced biomass production, which is consistent with the findings of Jisha et al. (2020) on centella. Moreover, the benefit of *S. indica* on biomass increase was significantly higher under WS than under NW, which may be related to the higher rate of fungal colonization formed by inoculated plants under WS conditions. On the other hand, *S. indica* also promoted host root growth by expanding the contact area between the root and the soil, which helped to improve the root’s ability to absorb water and nutrients, and thus improved growth (Li et al., 2020). In addition to growth improvement, *S. indica* colonization also significantly increased total chlorophyll content as *S. indica* improved nutrient uptake of host plant, such as Fe and Mg, which facilitate chlorophyll synthesis (Li et al., 2019), resulting in an increase in total chlorophyll of inoculated plants.

The present study showed that the WS treatment significantly increased the root H_2_O_2_ concentration, but decreased the root O_2_^•−^ concentration in inoculated and non-inoculated white clover. O_2_^•−^ is able to be scavenged by SOD and converted to H_2_O_2_ (Monika et al., 2021). The decrease in O_2_^•−^ concentrations and the increase in H_2_O_2_ concentrations in our study showed that *S. indica* initiated SOD to scavenge O_2_^•−^, thus inducing a burst in H_2_O_2_. Further analysis showed that inoculation of *S. indica* significantly reduced the H_2_O_2_ and O_2_^•−^ concentration of white clover under NW conditions, and only reduced the H_2_O_2_ concentration under WS conditions, with no significant effect on O_2_^•−^ under WS conditions, which is consistent with the results of previous studies on rice (Swetha and Padmavathi, 2019). This may be due to the fact that *S. indica* increased antioxidant enzyme activities and antioxidant concentrations in the plants, which synergistically scavenged excess ROS and thus reduced the oxidative damage to cells by ROS. As a result, the inoculated plants showed lower levels of MDA (an indicator of membrane lipid peroxidation).

Plants also possess antioxidant defense systems under stressful conditions to reduce ROS production, keep it at a relatively low level of ROS, and thus protect the structure of cell membranes (Swetha and Padmavathi, 2019). The results of this study showed that inoculation of *S. indica* under WS conditions did not alter root Mn-SOD activity, but significantly increased root Fe-SOD and Cu/Zn-SOD activities, suggesting that the alteration of host SOD by *S. indica* varied depending on the metal cofactors in SODs. In plants, Cu/Zn-SOD is predominantly in chloroplasts, cytoplasm, and peroxidase, while Mn-SOD and Fe-SOD are predominantly in mitochondria and chloroplasts, respectively (Du et al., 2001). This suggests that *S. indica* did not activate Mn-SOD activity in mitochondria under WS, but increased Fe-SOD and Cu/Zn-SOD activities in other organelles. In addition, the present study found that the plants inoculated with *S. indica* showed higher POD and CAT activities under NW conditions, suggesting that WS had an inhibitory effect on POD and CAT activities of white clover, which are the main enzymes for H_2_O_2_ scavenging (Du et al., 2001). Therefore, inoculated plants scavenged more H_2_O_2_, resulting in a decrease in H_2_O_2_. This is consistent with the findings of Khalid et al. (2018) on Chinese *Brassica napus* inoculated with *S. indica* under saline conditions. In addition to alterations in antioxidant enzyme activities, gene expressions of antioxidant enzymes are also modulated by *S. indica*. For example, *S. indica* induced *CsPOD* and *CsCAT1* expressions in leaves of Lane Late navel orange, together with an inhibition of *CsCu/Zn-SOD* expressions (Li et al., 2022b). The colonization of *S. indica* downregulated expressions of genes encoding enzymes for ascorbate synthesis in *Arabidopsis thaliana* (Vadassery et al., 2009). It suggests that antioxidant enzyme genes can respond to the colonization of *P. indica* at the molecular level, showing their important regulatory roles, and in-depth studies on the response pattern of antioxidant enzyme genes to *P. indica* are needed in the future.

The ascorbate-glutathione cycle in plants exists in the cytoplasm and chloroplasts and is an essential pathway to scavenge ROS (Liu et al., 2020). Ascorbic acid and glutathione in this cycle can maintain protein stability and cell membrane integrity, and together with APX and GR, constitute the ascorbate-glutathione cycle (Miao et al., 2021). GSH is a key indicator to determine the efficiency of the ascorbate-glutathione cycle (Ma et al., 2015). The results of this study showed that under WS condition, *S. indica* inoculation increased the activities of APX and GR in roots of white clover, and also promoted the concentration of ASC, GSH, and GSSG. These results suggested that the endophytic fungus *S. indica* could accelerate the ascorbate-glutathione cycle of white clover under WS to scavenge more ROS, thus alleviating the oxidative damage of WS on white clover.

## Conclusion

**Figure 7:**
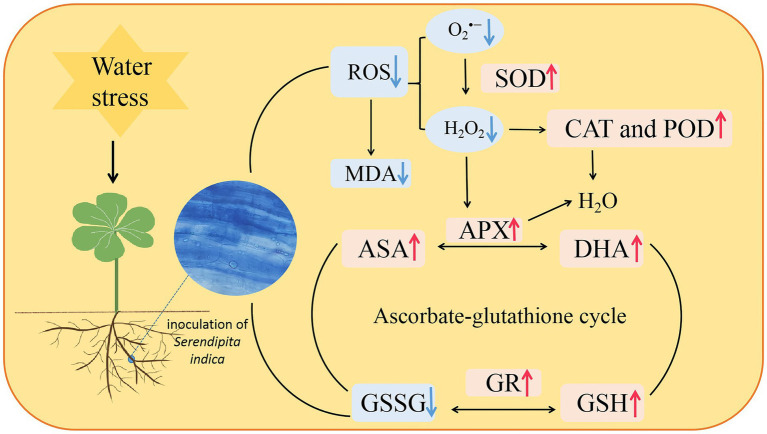
A mechanism diagram regarding changes in ascorbate-glutathione cycle in roots of white clover inoculated with *Serendipita indica* (Si) under water stress (WS). ↑, the parameter was significantly increased by Si; ↓, the parameter was significantly decreased by Si; ROS, reactive oxygen species; O_2_^•−^, superoxide anion; H_2_O_2_, hydrogen peroxide; SOD, superoxide dismutase; CAT, catalase; POD, peroxidase; MDA, malondialdehyde; APX, ascorbate peroxidase; ASC, ascorbic acid; DHA, dehydroascorbate; GSH, glutathione; and GSSG, oxydized glutathione.

In conclusion, the endophytic fungus *S. indica* was able to colonize white clover, and soil WS enhanced its colonization of roots. Colonization of roots by *S. indica* enhanced antioxidant enzyme activities and accelerated the ascorbate-glutathione cycle, which collectively scavenged ROS accumulation, thus maintaining low oxidative damage on inoculated plants under WS conditions (Figure 7). Such results provide a feasible idea for the future application of *S. indica* in the water-saving cultivation of white clover.

## Data availability statement

The original contributions presented in the study are included in the article/supplementary material, further inquiries can be directed to the corresponding author.

## Author contributions

Z-YR and Q-SW designed the experiment. J-LC prepared the materials for the experiment. Z-YR, D-JJ, J-LC, and Q-SW analyzed the data. Z-YR wrote the manuscript. AH, EFA, MA, WH, and Q-SW revised the manuscript. All authors contributed to the article and approved the submitted version.

## Funding

This work was supported by the Hubei Agricultural Science and Technology Innovation Action Project [Hubei Nongfa (2018) No. 1]. The authors are grateful to their sincere appreciation to the Researchers Supporting Project Number (RSP-2021/356), King Saud University, Riyadh, Saudi Arabia.

## Conflict of interest

The authors declare that the research was conducted in the absence of any commercial or financial relationships that could be construed as a potential conflict of interest.

## Publisher’s note

All claims expressed in this article are solely those of the authors and do not necessarily represent those of their affiliated organizations, or those of the publisher, the editors and the reviewers. Any product that may be evaluated in this article, or claim that may be made by its manufacturer, is not guaranteed or endorsed by the publisher.
